# Early Trauma and Cognitive Functions of Patients With Schizophrenia

**DOI:** 10.3389/fpsyt.2019.00261

**Published:** 2019-04-18

**Authors:** Carolina G. Carrilho, Simone S. Cougo, Tatiane Bombassaro, André Augusto B. Varella, Gilberto S. Alves, Sergio Machado, Eric Murillo-Rodriguez, Dolores Malaspina, Antonio E. Nardi, André B. Veras

**Affiliations:** ^1^Translational Research Group on Mental Health (GPTranSMe), Dom Bosco Catholic University, Campo Grande, Brazil; ^2^Research Laboratory on Autism and Behavior (LAPAC), Dom Bosco Catholic University, Campo Grande, Brazil; ^3^Department of Internal Medicine, Federal University of Maranhão (UFMA), São Luís, Brazil; ^4^Physical Activity Neuroscience Laboratory, Physical Activity Sciences Postgraduate Program-Salgado de Oliveira University (UNIVERSO), São Gonçalo, Brazil; ^5^Intercontinental Neuroscience Research Group, Universidad Anáhuac Mayab, Mérida, Mexico; ^6^Laboratory of Panic and Respiration (LabPR-UFRJ), Psychiatry Institute of Federal University of Rio de Janeiro (IPUB-UFRJ), Rio de Janeiro, Brazil; ^7^Laboratorio de Neurociencias Moleculares e Integrativas, Escuela de Medicina División Ciencias de la Salud, Universidad Anáhuac Mayab, Mérida, Mexico; ^8^Departments of Psychiatry, Neuroscience and Genetics, Icahn School of Medicine at Mt. Sinai Medical Center, New York, NY, United States

**Keywords:** schizophrenia, early trauma, cognition, memory, attention

## Abstract

**Aim:** The following work aims to investigate the putative correlation between early trauma and cognitive functions, as well as psychotic symptoms and cognitive functions, in individuals diagnosed with schizophrenia.

**Methods:** A quantitative assessment was performed with 20 individuals diagnosed with schizophrenia according to the 5th edition of the Diagnostic and Statistical Manual (DSM-5) criteria and who were in ongoing outpatient treatment in Psychosocial Care Centres in Brazil. Clinical measurements comprised a semistructured clinical interview, a screening questionnaire for common mental disorders, the Positive and Negative Syndrome Scale (PANSS), and the Early Trauma Inventory Self-Report—Short Form (ETISR-SF). Cognitive assessment included Beta III test, Concentrated Attention (CA) test, Color Trails Test (CTT), and Visual Face Memory (VFM) test.

**Results:** Age-adjusted analysis showed a negative correlation between early trauma and visual memory performance (r = −0.585, p = 0.007) and negative symptoms and attention performance (r = −0.715, p = 0.000).

**Conclusion:** Although a cause–effect relationship cannot be firmly stated, an association between early trauma experience and cognitive impairment such as visual memory, as well as a relationship between negative symptoms and attention domains, is suggested by our preliminary findings. Future studies with larger sample sizes and prospective design will clarify the long-term effects of early exposure to trauma and its clinical meaning in terms of developing psychotic-related illness.

## Introduction

More than 75% of patients with schizophrenia show some level of cognitive impairment, leading to poor functional status and impairments in social interaction. Therefore, when there is an improvement in cognition and executive function, there is also an improvement in quality of life, social interactions, and treatment outcomes (1).

Thus, cognitive performance in patients with schizophrenia may be related to the illness itself and to endophenotypes, with cognitive profiles indicating traits, which can be developed later on the course of the illness (2). This hypothesis has been supported by studies showing an overlap between cognitive deficits between SZ patients and their relatives. Patients with schizophrenia show a lower performance in neuropsychological tests when compared to both healthy controls and siblings. In addition, overlapping cognitive symptoms in patients with schizophrenia and their siblings seem to appear, with the latter exhibiting milder cognitive impairment, including memory, executive function, and attention.

Studies suggested that early trauma could be a risk factor associated with the development of schizophrenia. Environmental stimuli during childhood are crucial for the development of functional abilities in the human brain. Accordingly, physical or emotional trauma during childhood may cause cognitive impairment and increase the risk of developing schizophrenia or other mental disorders later in life (3). Early trauma is related to a reduced brain volume and reduced brain activity, therefore contributing to some of the neuropsychological impairments present in patients with schizophrenia. Moreover, patients with schizophrenia and an early trauma history show greater impairments in memory, attention, and social and executive functioning (4, 5).

Although previous studies suggest an association between early trauma and the risk to develop schizophrenia, there is still poor comprehension on how traumatic experiences may influence psychopathological and cognitive symptoms in those individuals. The current study is a preliminary investigation aiming to identify early trauma and their relationship with cognitive functions, and how cognitive functions can correlate to psychopathology in a clinical sample of patients with schizophrenia. We hypothesize that specific cognitive and psychopathological symptoms observed in schizophrenic individuals may be associated with early life exposure to sexual or psychological traumatic experience.

## Methods

All interviews and clinical assessments were conducted by one experienced psychiatrist (AV) and psychologists (SC and CC), no power analysis was carried out, and the recruitment was done by convenience with patients who were in ongoing outpatient treatment in Psychosocial Care Centres for mental health.

### Participants

Twenty individuals with a diagnosis of schizophrenia according to the DSM-V criteria (6) were assessed. Of the 20 subjects, 15 were male and 5 were female, with ages ranging from 23 to 54 years old.

Inclusion criteria included subjects from both sexes (male/female), with an age ranging from 18 to 65 years old and a diagnosis of schizophrenia according to the DSM-V criteria, who were on outpatient treatment in a Psychosocial Care Centre in Brazil, were able to understand the instructions provided by the interviewer in the assessment process and able to understand and sign the informed consent form (ICF), and have formal authorization by their legal guardians to participate in the research.

Subjects who did not meet the inclusion criteria and individuals at psychotic states or any other acute condition (e.g., disorganized mental activity or psychomotor agitation), with neurodegenerative problems that have compromised cognitive functions, as well as any other primary condition rather than schizophrenia (alcohol or substance abuse, mood, or anxiety disorders), were excluded from the study. Of the 20 subjects initially approached, all agreed to participate in the study and met the inclusion criteria.

### Materials

The research was approved by the local Ethics Committee. All patients answered sociodemographic data that were relevant to the research (age, ethnicity, education, occupational situation, family configuration, hospitalization history, and the development of schizophrenia). In addition, data were also supplemented with the information gathered from medical records of each patient or a complementary interview with the relatives.

#### Clinical and Psychiatric Measures

Clinical and psychiatric assessment comprised the following instruments: a) Screening Questionnaire [an instrument developed by the Psychiatry Genomics Consortium (PGC)—Centre of Genetic Psychiatry, from the University of Southern California and translated to Portuguese; the questionnaire screened for schizophrenia, mania, depression, use of substances, obsessive–compulsive behavior, post-traumatic stress symptoms (based on the DSM-5 criteria), and physical health history]; b) a Portuguese version of the Diagnostic Interview for Psychosis and Affective Disorder (DIPAD) (its objective is to use diagnostic criteria for schizophrenia and bipolar disorder through sociodemographic data and questions to identify depression, mania, and psychotic symptoms); c) Positive and Negative Syndrome Scale (PANSS) (7); and d) Early Trauma Inventory Self Report—Short Form (ETISR-SF) (8) composed of 27 items punctuated in dichotomous responses assessing the occurrence of trauma in childhood and adolescence. The assessment was subdivided into categories: general trauma (for example, occurrence of natural disasters, witnessing the death of close people, and separation of parents), physical abuse, emotional abuse, and sexual abuse.

#### Cognitive Assessment

Cognitive assessment included the following instruments: a) BETA III: Revised Beta Examination to assess intellectual abilities in low educated or illiterate subjects [nonverbal intellectual abilities through five subtests were explored: codes, complete figures, difference assessment, pictorial errors, and matrix reasoning (9)]; b) Visual Face Memory (VFM) [encompassing face memorization and other related information, e.g., names and surnames, profession, location, and employment (10)]; c) Concentrated Attention (CA; the test evaluates the ability to maintain focused attention by selecting a source of information among all that are available at a given time and manage to direct their attention to this stimulus during a certain time) (11); and d) Color Trails Test (CTT)—Forms A and B, which evaluates several functions, including the ability to maintain mental engagement, visual tracking, motor skills, mental flexibility, and inhibitory capacity. In Form A, the individual will draw lines by joining numbered circles consecutively, thus assessing sustained attention. In Form B, the individual will connect the circles with numbers consecutively, interspersing them with colors, thus assessing cognitive flexibility and divided attention (12).

### Data Analysis

Continuous variables were evaluated for their mean and standard deviation (SD), and categorical variables were evaluated for absolute and relative frequencies. Correlations were assessed using Spearman’s *r* test, considering statistically significant associations (those with a level of p ≤ 0.05). Continuous variables were sorted by cognitive tests such as BETA III, VFM, CA, and CTT, and psychopathological assessment such as general trauma, physical trauma, sexual trauma, negative PANSS, and general symptoms PANSS. Categorical variables were divided into sex, marital status, occupational situation, education, and ethnicity. Data were analyzed using SPSS 20.0 version package.

Furthermore, because of the hypothesis-based proof-of-concept nature of this analysis, correction for multiple testing was not applied. This is a pilot study that aims at hypothesis testing for future protocols. Therefore, in a small sample, when there is a prior understanding of the correlations intended in order to make a pilot study (hypothesis testing), statistical tests that are used for larger samples in fully conclusive clinical trials are not needed.

## Results

### Sociodemographic Characteristics

Research participants (n = 20) had a mean age of 37.4 years, were predominantly male (75%), were single (75%), were of African descent (60%), and were retired (80%). Most had a low education level (45% with incomplete primary education), with an average of 8.5 years in school (SD ± 3.14). Complete information on sociodemographic data is presented in **Table 1**.

**Table 1:** Sociodemographic profile of the sample.

Sex	N (%)
Male	15 (75%)
Female	5 (15%)
Marital status	N (%)
Single	15 (75%)
Married	3 (15%)
Widower	1 (5%)
Divorced	1 (5%)
Occupational situation	N (%)
Retired	16 (80%)
In the process of retirement	2 (10%)
Withdrawn from work	2 (10%)
Education	N (%)
Elementary school incomplete	9 (45%)
Complete primary education	1 (5%)
Incomplete high school	3 (15%)
Complete high school	6 (30%)
Higher education	1 (5%)
Average education in years of study	8.5 (SD ± 3.14)
Ethnicity	N (%)
Indigenous	2 (10%)
Caucasian	2 (10%)
Black	4 (20%)
Mulatto	12 (60%)

**Figure 1:**
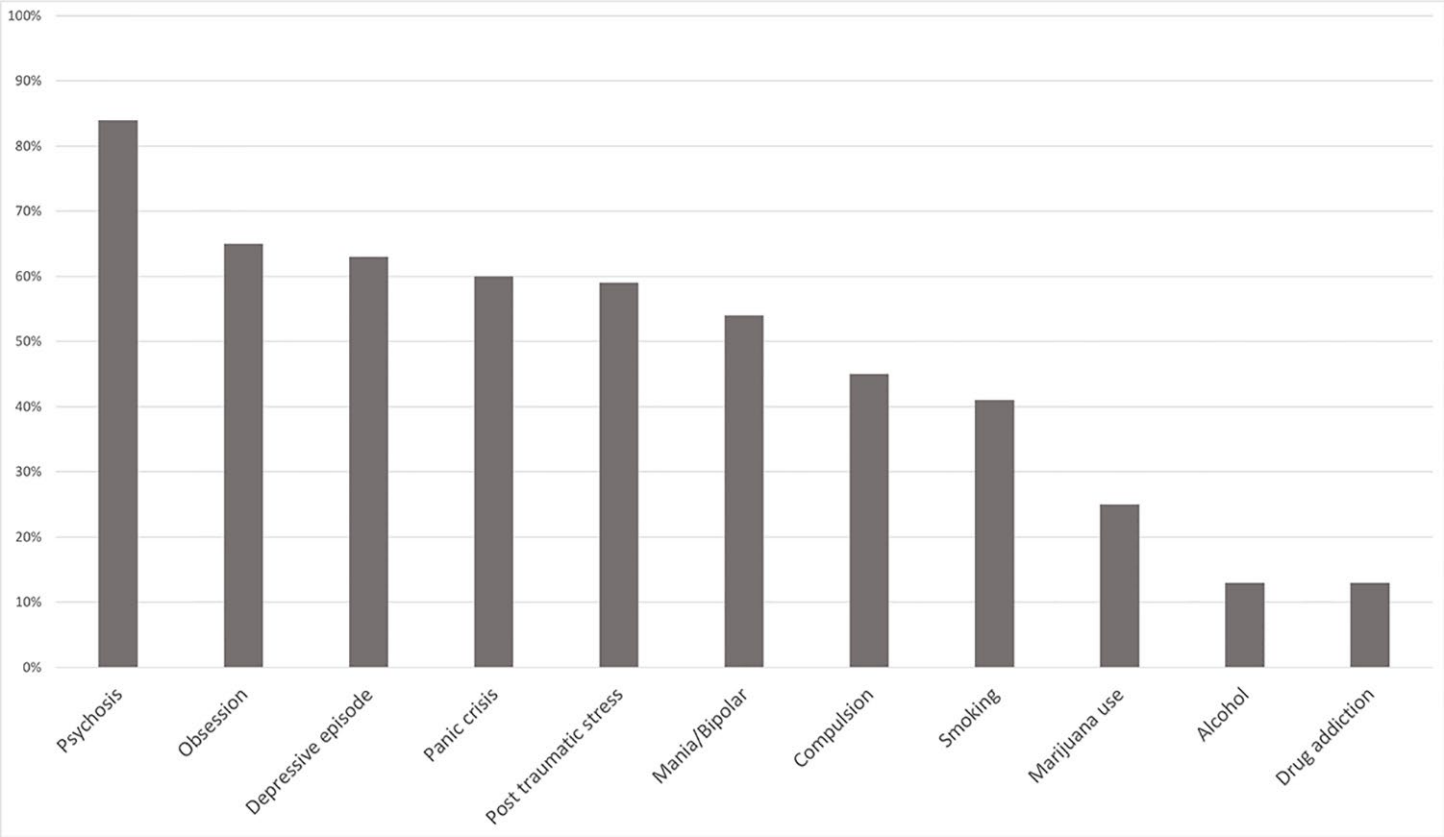
Mean of the positive scores of the Screening Questionnaire.

The arithmetic mean of percentages with a positive response in the symptoms proceeded from the Screening Questionnaire, and 84% of the participants reported psychotic symptoms, 65% had obsessive symptoms, 63% had symptoms of post-traumatic stress, 54% had symptoms of mania, 45% presented compulsion, 41% had tobacco use, 25% had use of marijuana, 13% had use of alcohol, and 13% had dependence of other drugs. The data are shown in **Figure 1**.

In the assessment with the Diagnostic Interview for Psychoses and Affective Disorders (DIPAD), it was noted that the average age of individuals at the onset of schizophrenia was 20.8 years (SD = 8.35). Of the 20 participants in the study, 11 participants (55% of the sample) were employed/studied at the time of the onset and 16 (80%) had good social relationships (family/friends) before the first crisis. In the subscale with questions about depression, 12 participants (60%) presented symptoms of depression. In the questions about mania symptoms, most participants (65%) reported no symptoms. In the subscale with questions about delusions, 17 participants (85%) reported having strange or unusual experiences—beliefs that they later found to be untrue. All participants (100%) reported some level of positive response to neuroleptic medications to control the positive symptoms—delusions and hallucinations—after initiation of drug treatment.

The mean of the total score of the participants for this instrument was 61.1 (SD ± 11.69), and the subscales presented the following results: positive symptoms presented a mean of 14.6 (SD = 11.69), negative symptoms presented a mean of 12.4 (SD = 4.57), and overall psychopathology presented a mean of 33.55 (SD = 6.19).

Of the 20 people assessed, only 1 reported not remembering to have suffered any traumatic situation in childhood, and 1 reported a situation linked to the general trauma subscale (parental divorce). Thus, 90% of the sample reported suffering at least five traumatic situations (**Table 2**).

**Table 2:** Results of the early trauma scale.

Type of trauma	Statistics	Value
General trauma	Mean	4.19
	Standard deviation	2.65
	Minimum	0
	Maximum	10
Physical punishment	Mean	2.24
	Standard deviation	1.87
	Minimum	0
	Maximum	5
Emotional abuse	Mean	2.62
	Standard deviation	1.96
	Minimum	0
	Maximum	5
Sexual events	Mean	1.38
	Standard deviation	1.94
	Minimum	0
	Maximum	6
Fear and out of body feeling	Mean	1.0
	Standard deviation	0.80
	Minimum	0
	Maximum	2
Total of items	Mean	11.10
	Standard deviation	6.75
	Minimum	0
	Maximum	22

In this instrument, the average of different types of trauma experienced by the sample was calculated according to the subscales and total scale. In the general trauma subscale, with issues involving exposure to accidents, illness, and deaths of close associates, an average of 4.19 positive responses were identified among the 11 dichotomous issues that could be scored. Considering physical punishment, emotional abuse, and sexual abuse, it was possible to observe the higher frequency of emotional abuse (mean of 2.62 out of five questions), physical punishment (2.24 of five questions), and sexual abuse (1.38 of six questions). The Fear and Out of Body Feeling subscales (altered state of consciousness) consist of trauma intensity markers.

### Cognitive Performance

**Figure 2:**
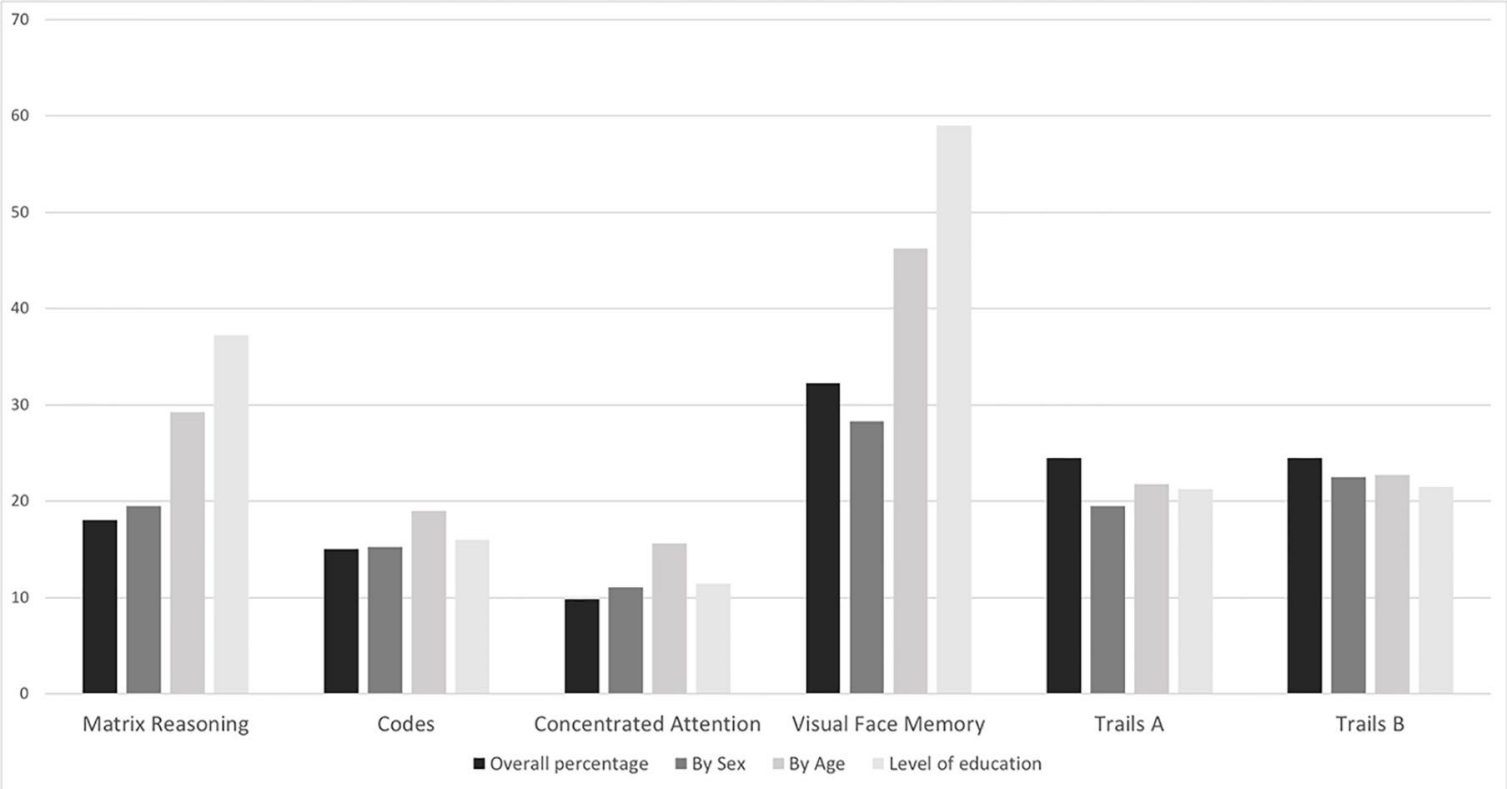
Mean of cognitive tests scores.

In the Matrix Reasoning (from BETA-III) subtest, the overall average was 18 (SD ± 8.8), corresponding to performance below the mean of the population assessed by the test, according to sex (average of 19%; median-inferior rating), age (29%; median-inferior rating), and level of education (37%; average rating; **Figure 2**). In the Codes—BETA III subtest, the overall mean was 15 (SD ± 10), corresponding to performance below the mean of the population assessed by the test, according to gender (average 15.25%; median-inferior rating), age (19%; lower rating), and level of education (16%; lower rating; **Figure 2**). The CA instrument presented an overall mean of 9.8 (SD ± 12), corresponding to performance below the average of the population evaluated according to the test references. Regarding gender, the result presented an average of 11% (medium-inferior rating), age 11.4% (medium-inferior rating), and level of education 15.6% (medium-inferior rating; **Figure 2**). The Visual Face Memory (VFM) test showed an average of 32.25 (SD ± 20.3), corresponding to performance within the average of the population, according to gender (average 46.25%; medium rating), age (28.25%; medium-inferior rating), and level of education (59%; medium rating; **Figure 2**). In the CTT—Form A, the result presented an average of 24.5 (SD ± 15.3), corresponding to performance below the average of the population, according to gender (average 21.75%; inferior rating), age (19.5%; inferior rating), and level of education (21.25%; inferior rating; **Figure 2**). In Form B of the CTT, the result presented an average of 24.5 (SD ± 11.7), corresponding to an inferior performance, according to gender (mean 21.75%; inferior rating), age (19.5%; inferior rating), and level of education (21.25%; inferior rating; **Figure 2**).

Correlations between early trauma (ETISR-SF) and VFM according to age and level of education, as well as a correlation between psychopathology (PANSS) and attention (AC and CTT), were found. The correlations observed between variables that showed a trend towards statistical significance are shown in **Table 3**.

**Table 3:** Correlations between cognition and trauma with the Early Trauma Inventory Self Report—Short Form (ETISR-SF), and between cognitive tests and the Positive and Negative Syndrome Scale (PANSS).

Cognitive tests ↓	Psychopathological assessment →	Overall trauma	Physical trauma	Sexual trauma	Total trauma	Negative PANSS	General symptoms PANSS	Total
CA (Concentrated Attention) (age)	r					−0.516		
	Sig.					0.02		
VFM (Visual Face Memory) (overall)	r				−0.516			
	Sig.				0.02			
VFM (Visual Face Memory) (age)	r	−0.446			−0.585			
	Sig.	0.049			0.007			
VFM (Visual Face Memory) (sex)	r				−0.444			
	Sig.				0.05			
VFM (Visual Face Memory) (level of education)	r			−0.597				
	Sig.			0.005				
Trails A (Color Trails Test) (overall)	r						−0.455	
	Sig.						0.44	
Trails A (Color Trails Test) (level of education)	r					−0.449	−0.542	−0.508
	Sig.					0.047	0.014	0.022
Trails B (Color Trails Test) (overall)	r					−0.631	−0.474	−0.515
	Sig.					0.003	0.035	0.02
Trails B (Color Trails Test) (age)	r					−0.715	−0.549	−0.604
	Sig.					0.000	0.012	0.005
Trails B (Color Trails Test) (sex)	r		0.454			−0.596		
	Sig.		0.045			0.006		
Trails B (Color Trails Test) (level of education)	r					−0.521	−0.622	−0.630
	Sig.					0.018	0.003	0.003

## Discussion

In this study, a negative correlation between general early trauma and visual memory (according to age and level of education) was found, that is, the higher the individual’s early trauma score, the lower the visual memory performance. Studies show that individuals with schizophrenia and a history of early trauma show memory deficits when compared to schizophrenia patients with no history of early trauma, and that these traumatic experiences may lead to neurocognitive deficits (13). A neuroradiological and neuropathological study conducted by Suddath et al. (14) with twins with schizophrenia concluded that anatomical changes in the brain, such as a smaller left anterior hippocampus, are present in almost every twin with schizophrenia and not present in healthy twins. This finding indicates that hippocampal deficits are related to schizophrenia.

Within the sample assessed in the present study, 90% of the individuals reported suffering trauma during childhood, with emotional abuse being the most frequent, followed by physical abuse and sexual abuse. Gil (15) noted that there is a great relationship between early adversities and increased vulnerability to psychosis. With a sample of 100 individuals diagnosed with schizophrenia, the author observed that traumatic experiences increase or reduce the vulnerability to psychotic disorder. Besides, in addition to influencing the etiology of schizophrenia, these traumatic experiences also affect the functional and social performance of the patient. Considering the studies mentioned, and the correlation found in the present study, nongenetic exposure such as early trauma can be an aggravating factor for the differences found in hippocampal activity (hippocampal deficit) in schizophrenic patients (13).

According to Lardinois et al. (16), exposure to trauma can affect dopaminergic transmissions and contribute to a hyperactivity of the hypothalamic–adrenal–pituitary axis (HPA axis) during childhood, which also increases sensibility to stress events in adulthood. Both of these phenomena can be found in individuals with schizophrenia and were not found in control groups. A systematic review conducted by Read et al. (17) with articles between 1972 and 2004 on schizophrenia and early trauma present the Traumagenic Neurodevelopmental Model hypothesis that when the trauma is prolonged, severe or daily, it can increase vulnerability to stress and contribute to a hypersensitivity that may lead the individual to be more prone to psychotic experiences. This indicates that neurologic and biochemical alterations found in schizophrenia and seen as etiological biogenetic evidence can also be related to early trauma and not only to genetic factors.

A study conducted by Veras et al. (18) that assessed the role of oxytocin in the pathogenesis of 48 patients with schizophrenia showed that in 5 of those cases, patients who presented a variation in oxytocin receptor rare single-nucleotide variants also showed more severe cognitive deficits, despite the severity of psychopathology, and a history of early trauma. Considering schizophrenia as a neurodevelopmental disorder, and since it is not a single-cause disorder and most commonly emerges in adolescence, any abnormal brain changes during adolescence such as the exaggeration of typical synaptic elimination and other neural elements may contribute to both the development of psychosis and impairment of cognitive functions later on (19).

We also observed a negative correlation between attention and negative symptoms, that is, the greater the presence of negative symptoms, the poorer the performance in attention (sustained and divided). Negative symptoms are usually related to attention impairments, and such impairments can cause disruption of the processing of social information. A study with 40 patients with schizophrenia and 40 healthy controls conducted by Sanz et al. (20) noted that patients with schizophrenia have severe attention deficits, and the lower the performance in attention assessment, the higher the score of negative symptoms in the PANSS scale. This indicates the patient’s inability to gate and process incoming information properly and that an impairment in attention can cause difficulty in interpersonal interaction (21).

Post-traumatic symptoms (59%), depressive episodes (63%), and panic attacks (60%) are within the most observed comorbidity symptoms in the sample. Buckley et al. (22) observed that there is a significant relation between different groups of symptoms in schizophrenia, with depression, anxiety, and substance abuse being the most common. Anxiety is commonly present in patients with schizophrenia, varying from OCD, panic, PTSD, social phobia, specific phobias, GAD, and acute stress disorder. Studies show a high prevalence of post-traumatic comorbid symptoms in schizophrenia, which should bring greater clinical attention due to the higher risk of suicidality of those individuals (23), and there is also an indication that treating comorbid anxiety can help in the course of schizophrenia (24).

A review (25) with studies published from 2001 to 2014 about the relationship between childhood trauma and symptoms of psychosis showed that the etiology of schizophrenia is just as socially based as disorders such as depression and anxiety, and childhood trauma causes changes in the brain involving the hippocampus and HPA axis that are also found in depression and post-traumatic stress disorder. Also, a research conducted in 100 patients with early psychosis showed that over three-quarters of those patients reported exposure to childhood trauma, and the childhood trauma scores were positively correlated with emotional distress, such as increased depression, anxiety, and stress symptoms (26).

Childhood trauma can be an aggravating factor for psychosis, since exposure to early trauma increases the risk of psychosis (27). A study conducted in three different hospitals in Oslo (28) assessed 194 patients with schizophrenia and noted that 82% of the patients had experienced some kind of childhood trauma, having a higher prevalence of childhood trauma when compared to other groups, such as control groups. There is also a correlation between childhood trauma and psychosis, since siblings of patients with schizophrenia present lower scores of abuse and neglect compared to patients with schizophrenia, which can indicate that exposure to trauma may result in illness (29). Some limitations of our study deserve further comments. First, the small sample size limits the power of statistical analysis. Second, there is an absence of a control group. Finally, the cross-sectional basis of our data precludes any firm conclusion on the cause–effect relationship between trauma and clinical features. In spite of these constraints, the correlations may be considered clinically significant enough to show a trend towards statistical significance even in a heterogeneous and naturalistic sample.

## Conclusions

This is the first study assessing a Brazilian sample on the relationship between early trauma, cognitive functions, and psychotic symptoms. Our preliminary findings, although limited by the small sample size, seem to validate the hypothesis of a specific association between memory deficits and childhood exposure to trauma. Our results thus shed light on the need to investigate the putative biological mechanisms related to the association of trauma and clinical characteristics in schizophrenia. Future studies encompassing larger samples and control groups are needed in order to address these questions with more details.

## Ethics Statements

Human Research Ethics Committee (Comite de Etica em Pesquisa de Seres Humanos - CEP) from the Dom Bosco Catholic University (Universidade Cat&amp;#243;lica Dom Bosco - UCDB).

## Author Contributions

CC and SC contributed to data collection and analysis, text review, and editing. TB contributed to data analysis, text review and editing, and tables editing. ABV and GSA contributed to data analysis, text review and mentoring. SM, EM-R, DM, AN and AABV contributed to text review and mentoring. CLA contributed to data design and critical review.

## Conflict of Interest Statement

The authors declare that the research was conducted in the absence of any commercial or financial relationships that could be construed as a potential conflict of interest.

The reviewer MS declared a past collaboration with one of the authors GA to the handling editor. This collaboration ended prior to the beginning of the reviewer's involvement in the review process.
