# Corrigendum: Early Trauma and Cognitive Functions of Patients With Schizophrenia

**DOI:** 10.3389/fpsyt.2019.00377

**Published:** 2019-05-28

**Authors:** Carolina G. Carrilho, Simone S. Cougo, Tatiane Bombassaro, André Augusto B. Varella, Gilberto S. Alves, Sergio Machado, Eric Murillo-Rodriguez, Dolores Malaspina, Antonio E. Nardi, André B. Veras

**Affiliations:** ^1^Translational Research Group on Mental Health (GPTranSMe), Dom Bosco Catholic University, Campo Grande, Brazil; ^2^Research Laboratory on Autism and Behavior (LAPAC), Dom Bosco Catholic University, Campo Grande, Brazil; ^3^Department of Internal Medicine, Federal University of Maranhão (UFMA), São Luís, Brazil; ^4^Physical Activity Neuroscience Laboratory, Physical Activity Sciences Postgraduate Program–Salgado de Oliveira University (UNIVERSO), São Gonçalo, Brazil; ^5^Intercontinental Neuroscience Research Group, Universidad Anáhuac Mayab, Mérida, Mexico; ^6^Laboratory of Panic and Respiration (LabPR-UFRJ), Psychiatry Institute of Federal University of Rio de Janeiro (IPUB-UFRJ), Rio de Janeiro, Brazil; ^7^Laboratorio de Neurociencias Moleculares e Integrativas, Escuela de Medicina División Ciencias de la Salud, Universidad Anáhuac Mayab, Mérida, Mexico; ^8^Departments of Psychiatry, Neuroscience and Genetics, Icahn School of Medicine at Mt. Sinai Medical Center, New York, NY, United States

**Keywords:** Schizophrenia, cognition, early trauma, memory, attention

In the original article, there was an error. We did not add an acknowledgement section, which is required in order to acknowledge a financial contribution we recently received.

A correction has therefore been made to the original article and the section **ACKNOWLEDGEMENTS** has been added:

“Sergio Eduardo de Carvalho Machado, was supported by a grant from the Carlos Chagas Foundation for the Research Support in the State of Rio de Janeiro (FAPERJ), Young Scientists from the State of Rio de Janeiro, E -26/203.295/2017.”

The authors apologize for this error and state that this does not change the scientific conclusions of the article in any way. The original article has been updated.

